# Parasite Presence Induces Gene Expression Changes in an Ant Host Related to Immunity and Longevity

**DOI:** 10.3390/genes12010095

**Published:** 2021-01-13

**Authors:** Marah Stoldt, Linda Klein, Sara Beros, Falk Butter, Evelien Jongepier, Barbara Feldmeyer, Susanne Foitzik

**Affiliations:** 1 Institute of Organismic and Molecular Evolution, Johannes Gutenberg University Mainz, 55128 Mainz, Germany; kllinda@students.uni-mainz.de (L.K.); foitzik@uni-mainz.de (S.F.); 2 Max Planck Institute for the Biology of Ageing, 50931 Cologne, Germany; Sara.Beros@age.mpg.de; 3 Institute for Molecular Biology, Johannes Gutenberg University Mainz, 55128 Mainz, Germany; F.Butter@imb-mainz.de; 4 Institute for Evolution and Biodiversity, University of Münster, 48149 Münster, Germany; e.jongepier@uva.nl; 5 Senckenberg Biodiversity and Climate Research Center (SBiK-F), Molecular Ecology, 60325 Frankfurt, Germany; barbara.feldmeyer@senckenberg.de

**Keywords:** host lifespan, transcriptomics, host–parasite interaction, *Temnothorax nylanderi*, *Anomotaenia brevis*, extended phenotype, Hymenoptera

## Abstract

Most species are either parasites or exploited by parasites, making parasite–host interactions a driver of evolution. Parasites with complex life cycles often evolve strategies to facilitate transmission to the definitive host by manipulating their intermediate host. Such manipulations could explain phenotypic changes in the ant *Temnothorax nylanderi*, the intermediate host of the cestode *Anomotaenia brevis*. In addition to behavioral and morphological alterations, infected workers exhibit prolonged lifespans, comparable to that of queens, which live up to two decades. We used transcriptomic data from cestodes and ants of different castes and infection status to investigate the molecular underpinnings of phenotypic alterations in infected workers and explored whether the extended lifespan of queens and infected workers has a common molecular basis. Infected workers and queens commonly upregulated only six genes, one of them with a known anti-aging function. Both groups overexpressed immune genes, although not the same ones. Our findings suggest that the lifespan extension of infected workers is not achieved via the expression of queen-specific genes. The analysis of the cestodes’ transcriptome revealed dominant expression of genes of the mitochondrial respiratory transport chain, which indicates an active metabolism and shedding light on the physiology of the parasite in its cysticercoid stage.

## 1. Introduction

Parasitism—life by exploiting resources from other living organisms—is one of the most fascinating life-history strategies in nature and used by the majority of species on Earth [1]. As a parasite’s fitness is often based on its ability to infect and potentially manipulate a host, selection has led to the evolution of diverse and sophisticated infection or transmission strategies of parasites. In particular, parasites with complex life cycles have developed a variety of strategies to facilitate the trophic transmission from the intermediate to the definitive host. They can achieve this by altering their host’s morphology, physiology, or behavior to their benefit [2]. However, not all changes in hosts observed upon parasite infection are the result of direct manipulation by the parasite; some alterations evolved as host defenses, others are simply the by-product of infection, benefitting neither parasite nor host [3,4]. Some intriguing examples for host alterations are found in insects that serve as intermediate hosts, such as the neotropical ant *Cephalotes atratus* when infected by the nematode *Myrmeconema neotropicum*. In addition to changes in behavior, infected ants also display a striking alternate morphology, a berry-red abdomen, which attracts birds, the definitive hosts [5,6]. Phenotypic alterations observed in infected insect hosts can include an extended host lifespan. For example, infected female *Tenebrio molitor* beetles, intermediate hosts of the rat tapeworm *Hymenolepsis disminuta*, show an increased survival of 40% [7]. A similar phenomenon is found in mice, where the parasitic nematode’s release of the molecule ES-62 extends the lifespan of this mammalian host by 70 days likely due to anti-inflammatory properties of the secretion [8]. Some parasites use secretions to manipulate their host’s behavior such as the hairworm *Paragordius tricuspidatus*, which elicits water-seeking behavior in its cricket host by releasing proteins of the Wnt family directly into the head [9,10]. Other parasites hijack the immune system like the parasitic wasp *Cotesia congregate*, which elicits an elevated octopamine level in the hemolymph of its host *Manduca sexta* similar to what is observed after an immune challenge [11,12]. Additional proposed methods of host manipulation include energetic drain by scavenging nutrients or the direct damage of organs by the parasite infecting host tissue [13].

As these manipulations often result in the death or at the very least in fitness reduction of the intermediate host, detection of parasites via innate immunity is important. Insect hosts, for example, have evolved diverse defense and evasion mechanisms including phagocytosis, nodule formation, the encapsulation and later melanization of parasites and behavioral defenses such as social immunity [14,15]. On the other hand, parasites evolved strategies to evade and/or suppress the immune responses of their hosts [16]. Thus, studying the differences between infected and non-infected individuals might not only assist in gaining a deeper understanding of immune responses of the host, but moreover shed light on possible strategies of the parasite to suppress or evade the hosts’ immune system. An intriguing example of a parasite infection altering the host’s phenotype is represented by our focal host-parasite system, the parasitic tapeworm *Anomotaenia brevis*, and its intermediate host, the cavity-dwelling ant *Temnothorax nylanderi*. Eggs of the cestode *Anomotaenia brevis* are transmitted from the definitive host, a woodpecker, to the intermediate ant host via ingestion of infected bird feces during the ants’ larval stage [17]. Within the ant, the cestode eggs probably first develop into oncospheres as shown in tapeworms with comparable life cycles like *Hymenolepis diminuta* [18]. The oncospheres then access the hemocoel by rupturing the gut using hooks where they develop into cysticercoids, a larval stage of the parasite. In fact, infected workers can be parasitized by 1–72 cysticercoids (Sistermans, pers. comm.) [19]. The complex life cycle of the parasite is completed when woodpeckers feed upon parasitized ant colonies that reside in acorns and sticks on the floor of Western European forests. The cysticercoids develop into adult cestodes, which then attach to the bird’s gut [20].

On average, 19% of the adult workers, but never the queen, are infected in parasitized colonies [19]. Parasite infection induces multiple alterations in the phenotype of the social host. Firstly, infected ants exhibit a strikingly light, less sclerotized cuticle compared to their brownish nestmates [21]. Secondly, the behavior of infected workers is altered. They show reduced activity and flight behavior and remain mostly in the center of the nest on the brood pile [21]. Moreover, they receive more care from their nestmates and are fed more often [19]. Lastly, the physiology of infected ants is affected too. For example, infected workers possess a higher reproductive potential than uninfected workers do, and they lay eggs when the queen is removed or dies [22,23]. Moreover, infected colonies raise a higher proportion of intercastes [19], that is worker-queen intermediates, maybe because young queens are smaller in size [24]. In addition, the survival of infected workers is increased compared to their uninfected nestmates [25]. Indeed, long-term analyses show that more than half of all infected workers survive at least three years such that their survival during this time span does not differ from that of queens, who can live for up to two decades in this species [26,27]. Still, it is currently unknown what the average and maximal lifespan of infected workers is, albeit anecdotal evidence reveals that they can become seven years old (personal communication A. Buschinger).

Previous work on brain gene expression in infected and uninfected workers of *T. nylanderi* revealed expression changes linked to the infection status of the individual and the colony [23]. These changes included the downregulation of genes, like actin, myosin, and tropomyosin, associated with muscular functions and the upregulation of a few genes from longevity pathways. No clear upregulation of immunity genes was found, most likely due to the tissue type investigated—the brain. Here we focus on transcriptomic shifts in the ant hosts’ abdomen, where the cysticercoids reside and which contains fat-body tissue, involved in the regulation of immunity and longevity in social insect queens [28]. Using transcriptomic data of abdomens from ants of different castes—the reproductive queens and the usually non-reproductive workers—and varying individual and colony-level infection status, we have the following objectives: Firstly, we want to identify genes that exhibit strong expression changes induced by infection and which may explain aspects of the phenotypic alterations observed in infected workers. Those differentially expressed genes could either represent defense mechanisms of the host against its endoparasite, for example by melanization, they could be by-products of infection such as a more active metabolism, or transcriptomic changes actively induced by the parasite to alter the hosts’ phenotype. Secondly, we are interested in whether the extended lifespans observed for both infected workers and uninfected queens have a common molecular underpinning. Upregulation of the same genes linked to longevity pathways could suggest that the parasite hijacks the ants’ phenotypic plasticity to induce the expression of queen genes as it was previously shown in a parasitized wasp [29]. Lastly, we will study gene expression in cestode cysticercoids themselves to provide first information on the parasites’ transcriptome, gaining insights into whether the parasite is potentially synthesizing and actively secreting proteins into the host. Looking at transcribed genes in the parasite can only be a first step, proteomic studies have to follow to reveal which types of proteins, how many are indeed released, and what their function could be. Nevertheless, our transcriptome study investigating gene expression both in the host and in the parasite will allow a deeper understanding of the molecular underpinnings of this host–parasite system.

## 2. Materials and Methods

### 2.1. Collection and Sampling of Ant Colonies

**Figure 1:**
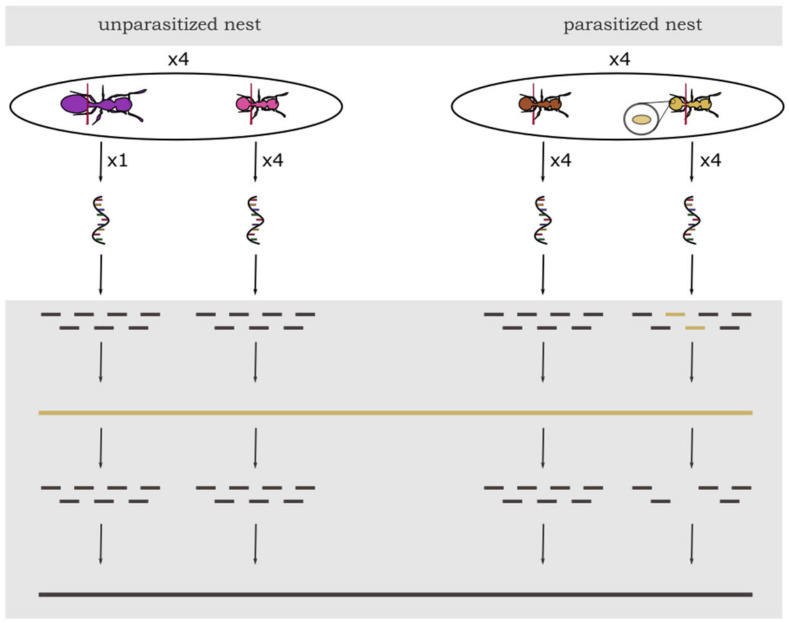
Experimental design of sampling and schematic analysis of gene expression data in *T. nylanderi* ants with different caste (workers or queens) and infection status. From each of the four unparasitized nests (left side) four workers were pooled per colony. Queen samples from unparasitized colonies consisted of only one individual. From each of the four parasitized nests (right side) four uninfected workers and four infected workers were pooled into two separate samples. Bottom grey part represents bioinformatic workflow: After sequencing, all samples were filtered for cestode RNA and other putative contaminants (yellow) and afterwards, all ant sequences were mapped against the genome of *T. nylanderi* for further analyses (black).

Ant colonies were collected in October 2017 and 2018 from the Lenneberg forest near Mainz, Germany. Ants were transported to the Johannes Gutenberg University Mainz, relocated to slide nests composed of a plexiglass cavity sandwiched between two microscope slides, and placed in three-chambered nesting boxes (see Supplementary Materials, Figure S1). Until sampling, the ants were kept at 22 °C and fed only with honey twice weekly, as feeding crickets could contaminate the gut content. Water was provided ad libitum. In November 2017, we selected four parasitized colonies and sampled four infected and four uninfected workers from the brood pile as separate groups. Infected workers are mostly found directly on the brood pile despite showing less brood care activity [19]. Infection status was preliminarily assessed by the color of the cuticle, which is yellow in infected workers in contrast to the typical brown color of their uninfected nestmates [21]. Important to note is that in parasitized colonies, infected workers can also occur with a normal brown cuticle, and without dissection or analysis of gene expression, infection status cannot be determined with certainty [19]. Therefore, we confirmed infection status later-on by mapping the reads of each sample against the cestode transcriptome. The gaster of the ants was detached directly in front of the postpetiole and four abdomens per group (infected workers and uninfected workers) were pooled into 100 µL of Trizol to reduce variance between samples. Abdomens were directly crushed in Trizol and RNA extracted using the RNAeasy mini extraction kit (Qiagen, Hilden, Germany) following the standard protocol. Sequencing of 75 bp single-end reads was performed on an Illumina NextSeq500 at the Institute of Molecular Biology (IMB, Mainz, Germany) in Mainz aiming for 15 Mio reads per sample to be able to detect strongly expressed genes. To investigate gene expression in queens in *T. nylanderi*, the entire procedure was repeated exactly one year later in 2018, with four unparasitized colonies (i.e., lacking yellow, infected workers) which did not differ in colony size from the parasitized ones (Wilcoxon, W = 3, *p* = 0.2). We sampled the only queen of each colony as well as four workers located directly on the brood pile whose abdomens were pooled again. With this, we were able to compare gene expression between queens and workers without the year of sampling as a confounding factor. As queens also usually reside on the brood pile, the location inside the nest was the same for all our four groups. This resulted in 16 samples (Figure 1, see Supplementary Materials, Table S6). Unparasitized colonies differed in size from 59–132 workers, indicating that queen age might also differ. *T. nylanderi* is a strictly monogynous species—meaning that colonies are founded and consequently headed by a single queen—so colony size is strongly linked to colony age [30].

### 2.2. Differential Expression Analysis in Ants

To remove all remaining cestode RNA and other putative contaminant sequences from all samples, we used FastQScreen (Babraham Institute, Cambridge, UK) with the newly assembled and filtered cestode transcriptome, sequences from humans, *Escherichia coli*, vectors, and adapters [31]. Detailed information about the assembly of the cestode transcriptome can be found in the Supplementary Materials. The infection status of worker pools from parasitized colonies was determined by identifying those four samples with a high percentage of cestode RNA (between 14 and 21%, see Supplementary Materials, Table S7).

RNA-Seq reads were trimmed using Trimmomatic (version 0.39) in single-end mode using the non-default trimming parameters: TRAILING 3, LEADING 3 and SLIDINGWINDOW 4:15 (see Supplementary Materials, Table S8) [32]. The quality of reads before and after trimming/filtering was assessed using FastQC [33]. Filtered reads were mapped against the genome assembly of *T. nylanderi* using HISAT2 v2.1.0 [34] with the parameter—dta as preparation for the genome-guided assembly. The resulting BAM files were sorted and indexed using Samtools v0.1.19 [35] and used to create a genome-guided transcriptome assembly using StringTie v1.3.6 [36]. To extract transcript sequences, we ran gffread v0.11.4 on the merged GTF file. We checked the transcriptome quality using TransRate v1.0.3 (see Supplementary Materials, Table S9) [37]. Predicted amino acid sequences of the transcripts were retrieved using TransDecoder v5.5.0 [38] and functionally annotated using InterProScan v5.46-81.0 [39]. The following analyses were conducted in R v3.6.1 [40]. We performed the differential expression analysis on the gene level and for this used the gene count matrix produced by StringTie. Thus, in the following “gene” refers to genes assigned by StringTie. To remove low read counts we only kept those genes that were mapped by at least five reads in at least three samples.

We conducted a Principal Component Analysis (PCA) using read counts for all genes and tested the association between infection status (infected/not infected) to each of the principal components (PC) using the Wilcoxon-test. For those PCs that significantly reflected differences in infection status (“infection PCs” hereafter), we identified top-loading genes and assessed their functional annotation. Hereto, the transcripts belonging to these genes were annotated using blastx v2.9.0 against the NCBI non-redundant invertebrate protein database (downloaded: 18.03.19), only considering hits with an E-value below 10^−5^ and taking for each transcript the hit with the highest bit score [41]. We assigned functions to genes that were represented by more than one isoform transcript by only considering the longest isoform (see Supplementary Materials). Enrichment analysis of the genes contributing to the “infection” PCs was performed using topGO v2.36.0 [42]. We used the weight01 algorithm and the Kolmogorov–Smirnov test to test for enrichment of functions of genes that were either positively or negatively associated with the “infection” PCs. By using the loading of each gene as a score, we were able to differentially weigh the functions of genes depending on their contribution to the PC. Only functions with *p* < 0.05 were considered as significantly enriched.

Gene expression was contrasted (1) between the infected and uninfected workers from parasitized colonies and (2) between queens and workers from unparasitized nests. For both comparisons we used Wald’s test implemented in DESeq2 v1.24.0 [43]. We only considered contigs with an adjusted *p*-value below 0.05 as significantly differentially expressed. The differentially expressed genes were checked for components of the melanization cascade in *Drosophila melanogaster* (see Supplementary Materials, Table S10) as we were interested in candidate genes for a potential immune response of the ant towards the cestode. Melanization is a common defense against endoparasites in insects such as *Drosophila melanogaster* and thus we decided to investigate especially genes contributing to the melanization cascade [44]. Functional enrichment of these differentially expressed genes (DEGs) was performed as described above only this time using a Fisher’s exact test to test for an overrepresentation of functions in our lists of overexpressed genes compared to all genes. In addition, we searched the BLAST hits of our differentially expressed genes against reviewed entries in the UniProt database from humans, mice, and *Drosophila* [45]. Afterward, we identified specific terms that are associated with longevity, fecundity, stress, and immunity (see Supplementary Materials, Table S11) in the lists of differentially expressed genes. We tested whether the groups differed significantly in the number of genes associated with these terms by using a χ^2^-test comparing the number of genes that are associated with this function to the number of genes that lack this functionality.

### 2.3. Expression of Cestodes in Ant Abdomens

In addition, we identified genes that were expressed by the cestodes inside the focal infected workers which were used for the analyses described above as these also included cestode tissue (see Section 2.2.). Therefore, we extracted reads from infected ants, which mapped one or multiple times against the newly assembled cestode transcriptome. Bowtie2 v2.3.5 [46] was used for mapping the filtered and trimmed reads against the cestode transcriptome and RSEM v1.3.1 [47] for transcript quantification to see which genes the cestodes express inside the ant’s abdomen.

## 3. Results

### 3.1. Gene Expression in T. nylanderi

#### 3.1.1. Principal Component Analysis

**Figure 2:**
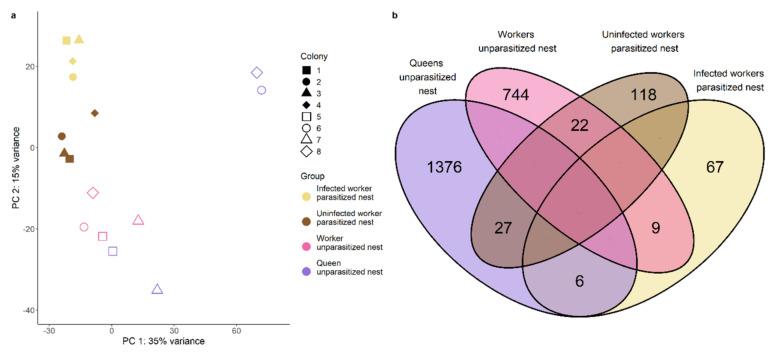
(**a**) Principal component analysis based on all genes. (**b**) Venn diagram depicting number of upregulated genes in pairwise comparisons between groups of the same nest and their overlap. Overlap, that is zero by default as the two groups compared do not share upregulated genes, is left blank.

**Figure 3:**
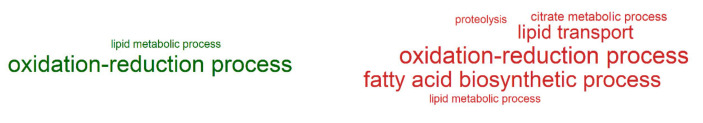
GO analysis of terms enriched in PC2 with positive loadings/positively correlated with PC2 (left in green) and negative loadings/negatively correlated with PC2 (right in red). Font sizes of individual terms are scaled by the negative natural logarithm of the *p*-value of the Kolmogorov-Smirnov test.

We sequenced the RNA of abdomens from infected and uninfected ants from parasitized nests as well as from queens and inside workers from unparasitized nests. Sequencing resulted in ~15 million 75 bp single-end reads per sample with an average GC content of 43.6% and a mean coverage between 13 and 18× (see Supplementary Materials, Table S8). These RNA reads were filtered for cestode RNA as cysticercoids are located in the ants’ abdomen. Moreover, we filtered the transcriptome to only contain transcripts with an open-reading frame at least 150 bp long and the gene count matrix for only those genes having at least 5 counts in at least 3 samples. After filtering, a total of 15,578 genes remained for further analysis. To gain insights into the significance of infection status on the general gene expression pattern, we conducted a PCA based on the overall read counts of these genes and tested the influence of infection status (infected or not) on each of the 16 principal components. Only PC2 and PC16 were found to be significantly influenced by infection status (Wilcoxon, PC2: W = 1, *p* = 0.002; PC16: W = 0, *p* = 0.001). As the latter explained less than one percent of the variance, we will focus on PC2 explaining 15% of the variance. Our samples clustered according to their group and infection status on PC2 (Figure 2a). Only the queens diverged from this pattern and showed the highest variance. As queen samples were derived from a single individual and not a pool of four individuals, it is not surprising they showed most within-group variance. While the queens from colonies 1 and 3 clustered near workers from the same nest, the queen samples from colonies 2 and 4 clustered between the infected workers and their nestmates. These latter two queen samples likely originated from older colonies, as their colonies contained more workers (117 and 132 workers vs. 59 and 76 workers). We were especially interested in genes contributing to PC2, where presumably older queens and infected workers clustered together, as these might correspond to age/longevity. Amongst the ten genes with the highest loadings on PC2, we found one encoding for *transferrin*, which is known to play a role in the innate immune system (see Supplementary Materials, Figure S2). The gene with the highest loading was encoding *bromodomain-containing protein 4-like isoform X1* in *Vollenhovia emeryi*, important in epigenetic memory in humans (UniProt). The enriched Gene Ontology (GO) terms in genes contributing mostly to PC2 in both directions were mostly linked to oxidation-reduction processes and metabolism (Figure 3). Genes negatively correlated to infection status were also enriched for fatty acid biosynthetic process and lipid transport.

#### 3.1.2. Differential Gene Expression Analysis

**Figure 4:**
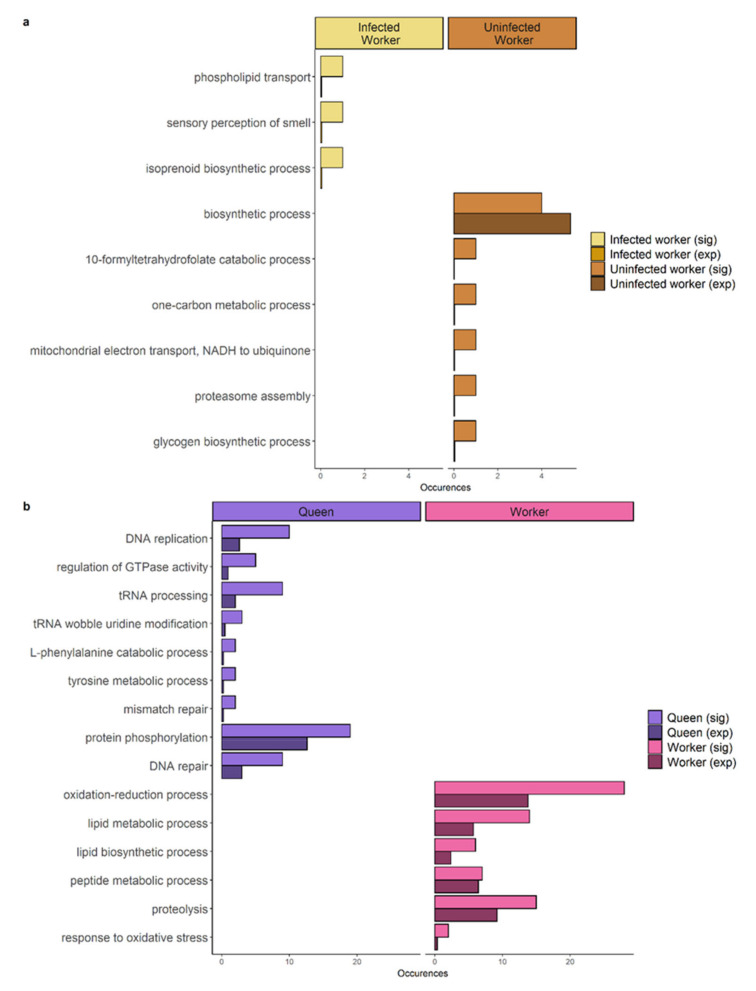
Bar plots depicting the significantly enriched Gene Ontology terms upregulated in (**a**) ants from parasitized nests: infected workers (left panel) and uninfected workers from parasitized nests (right panel). (**b**) ants from unparasitized nests: queens (left panel) and workers (right panel). Number of genes annotated with the specific term found in the candidate gene list are depicted (sig) as well as the number expected in a list of this size based on the annotation of the whole transcriptome (exp).

We found a total of 249 differentially expressed genes, when comparing gene expression between infected and uninfected workers from parasitized nests. Of those, 82 genes were upregulated in infected workers, while in contrast 167 genes were upregulated in uninfected workers. Amongst the ten genes upregulated in uninfected workers with the lowest adjusted *p*-value and a BLAST annotation, we found two genes encoding for vitellogenin receptors (see Supplementary Materials, Figure S3). In contrast, a gene encoding Mucin-2-like was found to be upregulated in infected workers (see Supplementary Materials, Figure S4 as well as Figures S5 and S6 for genes upregulated in the other groups). When looking at the annotations from UniProt, we identified amongst those genes two encoding proteins that had functional and/or GO annotations including melanization or the melanosome: carboxypeptidase B-like isoform X2 and lysosomal-trafficking regulator isoform X1. In infected workers, genes with functionality in isoprenoid biosynthetic processes and phospholipid transport as well as the perception of smell were enriched, yet these enrichments are based on single genes. Their uninfected nestmates showed upregulation of genes related to metabolism (Figure 4a).

Between queens and uninfected workers from the same nest, we found a total of 2184 genes to be differentially expressed, 1409 of them upregulated in queens and 775 in workers. Enriched functions in nurses from unparasitized nests were often stress-related, while queens upregulated more genes related to DNA replication and protein synthesis (Figure 4b).

**Figure 5:**
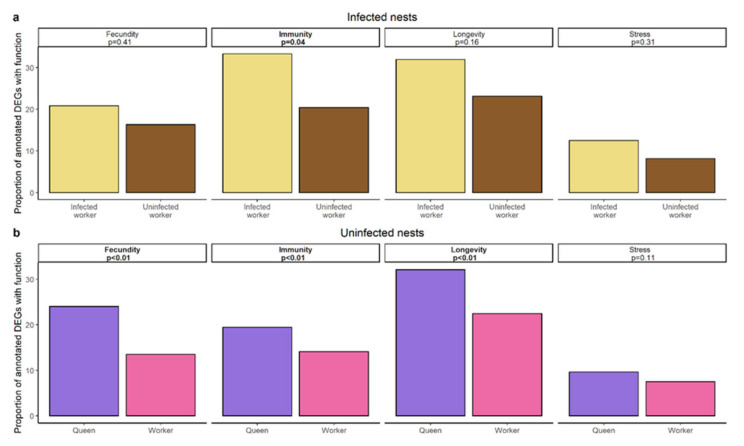
Results of text mining approach of terms related to fecundity, immunity, longevity, and stress based on a UniProt search of upregulated genes in (**a**) infected workers (yellow) and their uninfected nestmates (brown) of infected nests. (**b**) Queens (pink) and workers (purple) of uninfected nests. *p*-value of χ^2^-test and the according function are given in boxes.

The overlap between the genes differentially expressed between infected workers and their nestmates and the genes differentially expressed between queens and their workers comprised a total of six genes including one encoding carboxypeptidase B-like isoform X2 (Figure 2b, Supplementary Materials, Figure S7). Using a hypergeometric test based on the total number of differentially expressed genes this overlap was not significantly more than expected by chance (hypergeometric test: *p* = 0.309). Text mining for specific functionalities of DEGs revealed that infected workers upregulate more genes related to immunity than their uninfected nestmates (χ^2^ = 4.35, *p* = 0.037; Figure 5a) as queens (χ2 = 8.93, *p* = 0.003) do, in addition to genes related to longevity and fecundity (longevity: χ2 = 20.47, *p* < 0.00001; fecundity: χ2 = 30.79, *p* < 0.00001; Figure 5b). To ensure that the high variability of the queen samples (see Principal Component Analysis) does not lead to the detection of false positives, we repeated the gene expression analysis for queens and their workers, by taking colony size as a batch effect into account (see Supplementary Materials, Figures S8–S11). We compared those results to our previous analysis and focus our discussion only on those findings that are consistent between both analyses. In both analyses we only found little overlap between genes upregulated in queens and infected workers, the overlap in both cases comprising the gene encoding carboxypeptidase B-like isoform. Moreover, in both analyses we detected a strong representation of genes linked to immunity in infected workers and queens.

### 3.2. Cestode Transcriptome

We collected a total of 329 cysticercoids of the cestode *A. brevis* from 34 infected workers out of 18 different colonies, sequenced their RNA and assembled a first transcriptome using Trinity [38]. The final cestode transcriptome after all filtering steps consisted of 90,096 contigs with a mean length of 1079.01 bases and a GC content of 47%. Cestode specificity was confirmed as 94.65% of the contigs had best BLAST matches against other species from the phylum of Platyhelminthes. The overall alignment rate of the filtered and trimmed reads against the filtered transcriptome was 95.80%. Using BUSCO, we were only able to detect 24.1% of nematode orthologs in the cestode transcriptome (single-copy: 2.8%, duplicated: 21.3%, fragmented: 1.0%, missing: 74.9%) [48]. Analyzing the genes, which are highly expressed by the cestodes, we found that 97.89% of all reads originated from a gene encoding the cytochrome c oxidase subunit I, represented by in total 20 contigs (see Supplementary Materials, Table S5). Looking at the most prominent GO terms occurring in the cestode transcriptome, we did not find that those related to transport or specific metabolic networks were more prominent compared to the transcriptome of the ant (see Supplementary Materials, Figure S8). In addition, when analyzing the cestode transcripts from the ant abdomen (backmapping rate: 96.33–96.78%), we found cytochrome c oxidase subunit 1 (CO1) transcripts to be the most abundant, but a much smaller fraction of the reads was assigned to the CO1 gene (11.28–14.55%). Additionally, the subunits II and III were detected as the second and third most expressed transcripts in the transcriptome of the cestode as well as in the samples from the ant abdomens, but to a lesser extent (0.12–1.01%). The ten most expressed transcripts for each dataset are reported in the Supplementary Materials (Table S5).

## 4. Discussion

Interactions between parasites and hosts have led to fascinating cases of phenotypic alterations in hosts, many of them modulated to facilitate transmission to final hosts. Ants of *T. nylanderi* infected with the cestode *A. brevis* show a variety of morphological, physiological, and behavioral alterations compared to their nestmates. We analyzed the transcriptomic changes underlying these alterations to shed light on the molecular basis of the interactions between host and parasite. We observe gene expression changes in infected workers and propose three different not mutually exclusive causes for these: Firstly, a response of the host workers against the parasite. Secondly, they might indicate possible manipulation strategies of the parasite. Lastly, transcriptomic shifts might represent a mere by-product of parasite infection, for example, due to the higher physiological costs. By analyzing the cestode transcriptome, we provide first molecular data for this cestode, and identify putative mechanisms of host manipulation.

### 4.1. Infected Workers Upregulate Genes Involved in Immunity

Our study revealed genes related to immunity to be upregulated in infected workers compared to their nestmates. A previous study focusing on ant brains based on the same host–parasite system [23] found many differentially expressed genes in infected workers compared to uninfected ones, but no general overexpression of immune genes or genes similar to the ones overexpressed in our study. The difference is likely based on the different tissues, as the abdominal fat body is largely responsible for the immune response in insects [49]. As described above the observed changes in gene expression can be explained by multiple hypotheses that are not mutually exclusive: They might represent an immune response of the host workers against the parasite. Invertebrates including Hymenopterans largely rely on innate immune responses to defeat endoparasites—albeit there is some immune memory [14,50,51]. An important innate immune response is the encapsulation or melanization of parasites by hemocytes as seen in *Drosophila* flies parasitized by parasitoid wasps [44,52], which requires phenoloxidases or lectins [53,54]. The parasite, *A. brevis*, lives as cysticercoid larvae in the ant’s hemocoel, and thus might be directly threatened by encapsulation, melanization, or other immune defenses of the host [55]. Interestingly, dissections have so far revealed no evidence for an immune response towards the cestode, such as encapsulation or melanization. Of course, this does not rule out the possibility that certain hosts may successfully mount immune responses to inhibit or remove parasites. On the transcriptomic level, we found two genes upregulated in infected workers, which encode for proteins that have melanization functionalities. While carboxypeptidase is known to be involved in the melanization and immune response in *D. melanogaster* flies [56,57], the other candidate gene encoded a lysosomal-trafficking regulator, which also plays a role in immune defense [58]. Why did we find an upregulation of immune genes related to melanization when there is no histological evidence for such an immune reaction towards the cestode? One possible explanation could be the upregulation of multiple mucin-genes in infected workers. Mucins are known to be a host defense against helminths [59], and were also shown to protect larvae and eggs of parasitoids from being encapsulated by preventing the adhesion of hemocytes [60]. Upregulation of mucins in infected workers might indicate that parasite infection induces the production of mucins in the host, which in turn could prevent the encapsulation and melanization response. Although convergent evolution could occur, we rule out that these transcripts originate from the cestode itself as best BLAST hits of the mucins were found in ants, not cestodes and this was additionally confirmed by running blastn on the respective genes using default parameters against the nucleotide collection (nt) database. To investigate the link between mucin production and melanization response in hosts, further experiments are needed. These could include blocking mucin production in the host to test whether this results in encapsulation of the parasite. Furthermore, the observed changes in gene expression might represent a reaction not directed towards the cestode. Due to their lighter sclerotized cuticle, for example, infected workers might be more susceptible to other pathogens such as fungi and viruses, thus making an upregulated expression of immune genes necessary [61,62,63,64]. Our last proposed explanation for the underlying results in infected ants is a possible manipulation by the parasite. Activation of the immune system might be a route to modulate other characters of the ant by the cestode like behavior or morphology as shown for example in *Manduca sexta* [12,65]. Amongst the enriched functions, we found sensory perception of smell to be enriched in infected workers. In ants, perception is important for social behavior. In *Solenopsis invicta* for example, a single nucleotide polymorphism in a gene encoding an odorant-binding protein determines whether colonies accept only a single, or multiple queens [66]. Thus, the observed change in genes responsible for perception of smell might indicate a modulation of behavior. Whether this represents an active manipulation of behavior by the cestode through induction of the immune response or simply a by-product of infection, requires further testing. Additional studies could compare the expression of the corresponding gene in infected workers, infected workers with a killed cestode, immune-challenged workers and healthy workers. Moreover, future studies should aim for a higher sequencing depth to also detect more subtle gene expression changes.

### 4.2. Molecular Underpinnings of Longevity in Infected Workers and Queens

Infected workers in contrast to their uninfected nestmates show increased survival not different from that of the queens [25]. During their elongated life, infected workers are mostly inactive and do not contribute to colony life [19,67]. Infected ants do not reproduce in the presence of the queens and thus are unlikely to increase their direct fitness by living longer. Moreover, on a colony-level, the reduction in lifespan of uninfected workers in parasitized colonies likely outweighs the increase in lifespan of infected workers, since on average 19% of the workers are infected in parasitized colonies [19]. In addition, infected ants show a low activity level, contributing little to the usual worker chores such as brood care and foraging for food. By contrast, one may speculate that the longevity of infected individuals might be adaptive for the parasite. For the cestode to be transmitted to its final host, a woodpecker has to pick at the stick the colony resides in and pick-up an infected individual. Thus, the prolonged life of the ant host might increase the chances to be transmitted to the final hosts before the natural death of its intermediate host. Inducing the expression of queen-specific genes in infected workers would represents a cost-efficient strategy for the parasite and thus should be adaptive. If lifespan is manipulated by the parasite, we would expect infected workers to upregulate similar longevity-related genes as the queen. Thus, our first step was to characterize the transcriptomes of queens from unparasitized *T. nylanderi* colonies by contrasting them to uninfected workers from the same nest. This comparison showed the highest number of differentially expressed genes. In part, these transcriptomic differences might not only reflect variation in the regulation of fecundity and aging, but simply tissue composition in the gaster of queens and workers with queens exhibiting for example much larger ovaries relative to workers. Previous studies on *T. longispinosus* contrasting transcriptomes of queens and workers of different fertility status or developmental stages also showed highly divergent gene expression profiles in queens [68,69]. Moreover, queens of *T. longispinosus* upregulate genes with functions in DNA replication, which is similar to what we find here for *T. nylanderi* [69]. This is in accordance with comparative work showing that functionalities are conserved in castes of different species [70]. When looking at the overlap between genes upregulated in queens and infected workers, we found one promising candidate, the carboxypeptidase B, which in *D. melanogaster* is encoded by the silver gene and positively associated with increased lifespan [71]. However, generally speaking, we did not find more overlap than expected by chance in genes upregulated in infected workers with upregulated genes in queens. In part, this might be explained by differences in tissue composition and age between groups, which we could not control for as colonies were freshly sampled from the wild and experimental infection of nests is not established, or the fact that workers regulate longevity differently than queens. There is evidence for the latter in the closely related species *T. rugatulus* where fecund workers with extended life expectancy also seem to express different genes than queens [28,72]. Moreover, the lifespan extension of infected workers might be explained solely by social aspects. Infected workers were shown to be less active compared to their nestmates [19]. This might result in a lower production of reactive oxygen species and prolonged lifespan as shown in the housefly *Musca domestica* [73]. Additionally, since infected workers do not engage in foraging outside the nest, they have lower extrinsic mortality similar to queens which also stay inside the nest [19,74,75]. As a second line of evidence, we investigated genes with a high contribution to PC2, which grouped the two older queens with the infected workers and thus is linked to longevity. Two of the top genes seem to be involved in immunity. The gene with the highest contribution was encoding a bromodomain-containing protein, an epigenetic reader (Supplementary Materials, Figure S2), with a role in inflammation and cancer in humans [76]. Transferrin is a key player in iron metabolism, and previous work in insects suggests that it is also involved in immune response [77]. It was also one of the few genes involved in immunity found to be upregulated in the brains of infected workers [23]. In general, both queens and infected workers expressed more genes related to immunity than their nestmates. Immunity and longevity are tightly interlinked, but which of the two represents the cause or consequence of the prolonged lifespan remains to be investigated [78,79].

### 4.3. Overexpression of Cytochrome C Oxidase Subunit I in the Cestode

Overall, our newly assembled transcriptome of the cestode in the cysticercoid stage only contained 24% of genes usually found in nematodes, the phylogenetically closest phylum with an ortholog set available. This is to be expected as during this life stage only a part of genes is expressed. Moreover, the parasitic lifestyle itself might also result in gene loss and thus genome reduction as shown in other systems [80,81,82]. When analyzing the transcriptome of the cestode *A. brevis*, we found that the majority of reads belong to a single gene, encoding the *cytochrome c oxidase subunit I*, which represents the terminal part of the mitochondrial respiratory electron transport chain producing ATP. The strong overexpression of a single gene might additionally explain the observed incompleteness of our assembled transcriptome, leaving less coverage for other genes. We propose different non-exclusive explanations for such a strong overexpression of this gene: (1) Energy metabolism. Studies on other tapeworms show that the main energy resource is carbohydrates including trehalose and glycogen, which are absorbed from the hemolymph of the host and metabolized by aerobic respiration [81,83]. Moreover, preliminary data suggest that the parasite releases proteins into the abdomen of the ant, and for these transport processes, energy is required, which might be acquired using aerobic respiration (Butter, pers. comm.). (2) Prolongation of cysticercoid lifespan. In *C. elegans*, overexpression of cytochrome c oxidase was observed in dauer larvae as well as in long-lived adults [84]. The cysticercoid stage resembles in part the dauer state of nematodes like *C. elegans*. As the duration of the life stage inside the intermediate host can vary, cysticercoids might overexpress genes related to lifespan extension during this waiting period. Furthermore, the expression of CO1 is positively linked to longevity as the expression of cytochrome c oxidase declines as a function of age and in *Drosophila* this decline in expression can cause a shortened lifespan [85,86]. (3) Stress response. Cytochrome c oxidase is involved in an organism’s response towards oxidative stress probably via the handling of reactive oxygen species (ROS) as dysfunction causes an increase in ROS [87]. During dissections, the removal from their intermediate host might have triggered oxidative stress in cysticercoids, which resulted in the observed overexpression of CO1 to be able to cope with it. Moreover, oxidative stress could be the result of high ROS production in the host associated with an immune response, as shown in *D. melanogaster* [88]. (4) Exposition to oxygen. Due to our dissection protocol cysticercoids were exposed to a high level of oxygen compared to their previous environment inside the ant. Cytochrome c oxidase catalyzes electron transport towards molecular oxygen and its activity is correlated with oxygen levels [89]. Thus, the extreme upregulation of CO1 might be due to our dissection procedure which exposed cestodes to an unnaturally high oxygen level. Usually, the cysticercoid is removed from the ant when eaten by their final host, and exposure to oxygen might signal to the cestode that it needs to progress into its next life stage. Thus, overexpression of CO1 might be adaptive in the context of metamorphosis and establishment in the final host.

## 5. Conclusions

We were able to explain some of the physiological differences between infected workers and their nestmates through the expression of certain genes, including candidate genes related to melanization and immunity. Although there was only minimal overlap in genes upregulated in infected workers and queens, some of these genes are known to be involved in longevity in other insects and thus represent interesting candidates for future studies. Characterization of the cestode transcriptome revealed a strong expression of a gene involved in mitochondrial electron transport, indicating a high energy consumption by the parasite. We are currently conducting proteomic studies to investigate whether they can explain the elongated lifespan of infected workers. Moreover, we hope that in the future a sequenced genome will be available for *A. brevis*, which will allow us to gain more insights into the molecular underpinnings of the host-parasite interaction.

## Data Availability

The data presented in this study are openly available in Mendeley Data at doi:10.17632/7jvdd7jwks.1. Additional tables and figures referenced in the manuscript as well as detailed methods regarding the assembly of the cestode transcriptome. Additionally, the count matrix, the list of DEGs together with their UniProt annotation, the results of the GO enrichment, the transcriptomes, and the analytic scripts are provided as Supplementary Material separately. Raw reads can be accessed from SRA under BioProject ID PRJNA673150.

## Supplementary Materials

The following are available online at https://www.mdpi.com/2073-4425/12/1/95/s1, Figure S1: Expression of top four genes contributing to PC2 in the Principal Component Analysis, Figure S2: Top ten genes according to adjusted *p*-value that had a functional BLAST annotation upregulated in uninfected nestmates compared to infected workers of the same nest, Figure S3: Top ten genes according to adjusted *p*-value that had a functional BLAST annotation upregulated in infected workers compared to their uninfected nestmates, Figure S4: Top ten genes according to adjusted *p*-value that had a functional BLAST annotation upregulated in nurse compared to queens, Figure S5: Top ten genes according to adjusted *p*-value that had a functional BLAST annotation upregulated in queens compared to nurses, Figure S6: Genes overlapping between DEGs upregulated in queens and infected workers, Figure S7: Ten most prevalent GO terms found in the cestode transcriptome and the ant transcriptome, Figure S8: Overlap between differentially expressed genes upregulated in queens after controlling for colony size as batch effect and the genes upregulated in infected workers, Figure S9: Genes and their expression in overlap between differentially expressed genes upregulated in queens after controlling for colony size as batch effect and the genes upregulated in infected workers, Figure S10: Results of text mining approach of terms related to fecundity, immunity, longevity and stress based on a UniProt search of upregulated genes when controlling for colony size as batch effect, Table S1: Sampling information for *Anomotaenia brevis* cestodes, Table S2: Databases used for filtering of cestode RNA reads, Table S3: Read Statistics for *A. brevis*, Table S4: Assembly statistics for the filtered *A. brevis* transcriptome, Table S5: Top 10 expressed genes in the transcriptome of intact *Anomotaenia brevis* cestode pool as well as their proportion in the cestode transcripts from infected workers abdomens, Table S6: Colony information for colonies used for RNA-Seq samples of *T. nylanderi*, Table S7: Sample information for RNA-Seq samples of *T. nylanderi*, Table S8: Number of reads for samples of *T. nylanderi* before and after filtering and trimming, Table S9: Assembly statistics for the transcriptome of *T. nylanderi*, Table S10: Proteins involved in the melanization pathway in *Drosophila melanogaster*, Table S11: Search terms used for word search analysis based on the functional annotation using the UniProt database.
