# Supplementary material for: Parasite Presence Induces Gene Expression Changes in an Ant Host Related to Immunity and Longevity
Source: Genes (Basel). 2021 Jan 13;12(1):95. doi: 10.3390/genes12010095 (PMC7828512; doi:10.3390/genes12010095)
Supplement: Supplementary file 1 [file genes-12-00095-s001.pdf]

Supplementary Information

Parasite presence induces gene expression changes in an ant host and their function in immunity and longevity

Marah Stoldt[1], Linda Klein[1], Sara Beros[2], Falk Butter[3], Evelien Jongepier[4], Barbara Feldmeyer[5], Susanne Foitzik[1]

[1]Institute of Organismic and Molecular Evolution, Johannes Gutenberg University Mainz, Germany

[2]Max Planck Institute for the Biology of Ageing, Cologne, Germany

[3]Institute for Molecular Biology, Mainz, Germany

[4]Institute for Evolution and Biodiversity, University of Münster, Münster, Germany

[5]Senckenberg Biodiversity and Climate Research Center (SBiK-F), Molecular Ecology, Frankfurt, Germany

Materials & Methods

Collection and Sampling of Cestodes

Parasitized colonies of the ant *T. nylanderi* were collected in the years 2013 - 2015 in the Lenneberg forest and Eltville near Mainz, Germany. In January 2016, 40 infected workers from 18 different colonies were dissected, resulting in a total of 329 isolated cysticercoids that were collected and washed three times in DNase-free water to remove remains of ant tissue. RNA extractions were performed in four rounds so that dissections of cestodes for one round did not last longer than one hour (see Supplementary Information Table S1). Per round, cestodes were transferred into 100 µl of Trizol, and RNA extraction was performed using the RNAeasy mini extraction kit (Qiagen) and following standard protocol. Afterwards, samples were combined into a single pool and sent to BGI Hongkong for RNA sequencing on an Illumina HiSeq 4000.

Cestode Transcriptome Assembly

Sequencing resulted in 30 million 100 base pair (bp) paired-end reads with a GC content of 44 %. We could not rule out that ant cells remained on the surface of the cestodes after washing. To ensure that our samples contained no contaminant RNA, we used FastQScreen v0.14.0 to filter out non-cestode reads (Wingett and Andrews, 2018). To do so we used multiple genomes and databases (downloaded from the National Center for Biotechnology Information (NCBI) Genome Browser, see Supplementary Information Table S2) together with the genome assembly of the ant host against which the reads were mapped using Bowtie2 v2.3.5 (Langmead and Salzberg, 2012). Only those reads which did not map to any of the genomes or databases were taken for further analysis, representing 92.34 % of both the forward and the reverse reads (see Supplementary Information Table S3). The filtered reads were quality and adapter trimmed using Trimmomatic v0.39 (Bolger et al., 2014) with the quality trimming parameters LEADING 3, TRAILING 3, and SLIDINGWINDOW 4:15 (see Supplementary Information Table S3). The paired forward and reverse filtered and trimmed reads were used for *de novo* assembly using Trinity v2.8.4 (Haas et al., 2013) in forward-reverse mode specifying a minimum contig length of 300 bp. Assembly metrics were assessed using TransRate v1.0.3 (Smith-Unna et al., 2016) and

completeness of the transcriptome was assessed using BUSCO v4.1.2 with the nematoda_odb10 ortholog database on the European Galaxy Server (Afgan et al., 2018; Simão et al., 2015). This database was chosen as no ortholog set is yet available for cestodes and the nematode database was phylogenetically the closest. Species-specificity was ensured using the Basic Local Alignment Search Tool (BLAST) (Altschul et al., 1990). Hereto, and to assess the possible functionality of genes encoded by the cestode, we used the blastx algorithm against the invertebrate protein database (E-value cut-off 10^{-5}). Contigs matching sequences from Hymenoptera were filtered out to ensure no contamination with ant RNA, leaving the transcriptome assembly at a size of 90,096 contigs (see Supplementary Information Table S4). These transcripts were translated using TransDecoder v5.5.0 and used as input for InterProScan v5.46-81.0 (Jones et al., 2014). Read counts were assessed using Bowtie2 v2.3.5 (Langmead and Salzberg, 2012) to map the filtered and trimmed reads against the transcriptome and RSEM v1.3.1 (Li and Dewey, 2011) for transcript quantification (see Supplementary Information Table S5).

Additional gene expression analysis of queen and worker samples

We found that the queen samples show a high variability in the Principal Component Analysis, which we attributed to colony age. To ensure that this variability did not cause an inflation of false positives amongst our differentially expressed genes, we additionally performed the comparison between queens and their workers taking colony size as a batch effect into account. The results of the gene expression analysis are also provided in the Supplementary Material (see Supplementary Information Figure S8, S9, S10 and S11). In the manuscript only those results are discussed, which are robust in both analyses.

Table S1 Sampling information for *Anomotaenia brevis* cestodes.

	Cestodes Round 1	Cestodes Round 2	Cestodes Round 3	Cestodes Round 4
KLE14_004	8	3	1	
KLE14_021		10	23	
KLE14_029		4	24	14
KLE14_032		10		
KLE14_009		3	15	10
KLE13_HL058	5		5	
KLE13_HL026		9	3	
KLE13_HL021	10			19
KLE14_018	4			
KLE14_042	11	5		
KLE14_038	24	4		
LBW13_FS019		15		
LBW13_FS056		3	6	
LBW15_Q77	2	5	3	23
NRT 013				11
KLE14_NOV_033				16
KLE14_NO_004				8
KLE14_NO_042				13
**Total**	**64**	**71**	**80**	**114**

Table S2 Databases used for filtering of cestode RNA reads.

Species/ Sequence origin	Version	Obtained from
Escherichia coli	ASM584v2	Ensembl

Vectors	build 10.0	UniVec
Adapters	v0.11.8	FastQC
Homo sapiens	GRCh38	Ensembl
Temnothorax nylanderi	v1.0 (14.03.2019)	NCBI Genome Browser
Temnothorax rugatulus	v1.0 (05.03.2019)	NCBI Genome Browser
Temnothorax curvispinosus	ASM307098v1	NCBI Genome Browser
Solenopsis invicta	Si_gnH	NCBI Genome Browser
Wasmannia auropunctata	wasmannia.A_1.0	NCBI Genome Browser
Vollenhovia emeryi	V.emery_V1.0	NCBI Genome Browser
Monomorium pharaonis	ASM326058v2	NCBI Genome Browser
Trachymyrmex zeteki	Tzet1.0	NCBI Genome Browser
Apis mellifera	Amel_HAv3.1	NCBI Genome Browser
Drosophila melanogaster	Release 6 plus ISO1 MT	NCBI Genome Browser

Table S3 Read Statistics for *A. brevis*.

	**Forward**	**Reverse**
Raw	30610634	30610634
After Filtering	28265334	28264767
After Trimming	28260185	28260185

Table S4 Assembly statistics for the filtered *A. brevis* transcriptome.

**Parameter**	**Value**
Number of transcripts	90096
Mean length	1079.01
GC%	47
N50	1520

Complete BUSCOs %	24.1
Single-copy BUSCOs %	2.8
Duplicated BUSCOs %	21.3
Fragmented BUSCOs %	1.0
Missing BUSCOs %	74.9

Table S5 Top 10 expressed genes in the transcriptome of intact *Anomotaenia brevis* cestode pool as well as their proportion in the cestode transcripts from infected workers abdomens. Percentages in bold indicate the gene with the highest read counts for the respective sample.

	Read count attributed to each gene (%)				
**Protein encoded by gene**	**Pool intact cestode**	**Abdomen Pool 1**	**Abodomen Pool 2**	**Abdomen Pool 3**	**Abdomen Pool 4**
cytochrome c oxidase subunit I (mitochondrion)	**97.89 %**	**11.28 %**	**12.8 %**	**14.55 %**	**14.32 %**
cytochrome c oxidase subunit II (mitochondrion)	0.17 %	0.61 %	0.65 %	1.01 %	0.48 %
cytochrome c oxidase subunit III (mitochondrion)	0.12 %	0.58 %	0.62 %	0.7 %	0.74 %
Fatty acid-binding protein	0.1 %	0.37 %	0.41 %	0.39 %	0.4 %
Heat shock cognate protein	0.1 %	0.39 %	0.34 %	0.32 %	0.35 %
Succinate dehydrogenase	0.07 %	0.5 %	0.55 %	0.5 %	0.53 %
1,5-anhydro-D-fructose reductase	0.02 %	0.43 %	0.44 %	0.44 %	0.36 %
hypothetical protein EGR_08712	0.02 %	0.1 %	0.11 %	0.11 %	0.1 %
Elongation factor 1-alpha	0.02 %	0.4 %	0.43 %	0.38 %	0.39 %
NADH dehydrogenase subunit 5 (mitochondrion)	0.02 %	0.36 %	0.41 %	0.49 %	0.35 %

Table S6 Colony information for colonies used for RNA-Seq samples of *T. nylanderi*.

ColonyID	Queen	Uninfected workers	Infected workers
LBW17_A02	1	72	9
LBW17_A11	1	45	5
LBW17_A45	1	113	4
LBW17_A07	1	51	6
LBW18_K073	1	59	0
LBW18_K067	1	117	0
LBW18_K034	1	76	0
LBW18_K062	1	132	0

Table S7 Sample information for RNA-Seq samples of *T. nylanderi*. Four worker samples from infected colonies contained an extremely low percentage of reads (<1%) mapping to the cestode transcriptome that are no different from samples from uninfected colonies and were thus considered uninfected. Group affiliations were confirmed by testing whether the uninfected nestmates of infected nests differ in the amount of cestode RNA found from samples from uninfected nests (Wilcoxon, W=8, p=0.2). The amount of cestode RNA differed significantly between infected workers and uninfected workers from both infected and uninfected nests (Wilcoxon, W=0, p=0.004).

**Colony ID**	**Old Sample Name**	**Sample Name**	**Caste**	**Tissue**	**Individuals pooled**	**% cestode RNA**
LBW17_A02	1_4y_Abd_RNA_Replicate_1	Infected 1	Infected	Abdomen	4	18.83
(LBW17_A11)	2_4y_Abd_RNA_Replicate_2	Nestmate 1	Nurse	Abdomen	4	0.12
LBW17_A45	3_4y_Abd_RNA_Replicate_3	Infected 2	Infected	Abdomen	4	21.88
(LBW17_A07)	4_4y_Abd_RNA_Replicate_4	Nestmate 2	Nurse	Abdomen	4	0.09
(LBW17_A02)	1_4b_Abd_RNA_Replicate_1	Infected 3	Infected	Abdomen	4	18.24
LBW17_A11	2_4b_Abd_RNA_Replicate_2	Nestmate 3	Nurse	Abdomen	4	0.13
(LBW17_A45)	3_4b_Abd_RNA_Replicate_3	Infected 4	Infected	Abdomen	4	14.29
LBW17_A07	4_4b_Abd_RNA_Replicate_4	Nestmate 4	Nurse	Abdomen	4	0.15
LBW18_K073	1_4n_Abd_RNA_Replicate_1	Nurse 1	Nurse	Abdomen	4	0.22
LBW18_K067	2_4n_Abd_RNA_Replicate_2	Nurse 2	Nurse	Abdomen	4	0.24
LBW18_K034	3_4n_Abd_RNA_Replicate_3	Nurse 3	Nurse	Abdomen	4	0.24
LBW18_K062	4_4n_Abd_RNA_Replicate_4	Nurse 4	Nurse	Abdomen	4	0.13
LBW18_K073	1_1q_Abd_RNA_Replicate_1	Queen 1	Queen	Abdomen	1	0.12
LBW18_K067	2_1q_Abd_RNA_Replicate_2	Queen 2	Queen	Abdomen	1	0.19
LBW18_K034	3_1q_Abd_RNA_Replicate_3	Queen 3	Queen	Abdomen	1	0.11
LBW18_K062	4_1q_Abd_RNA_Replicate_4	Queen 4	Queen	Abdomen	1	0.14

Table S8 Number of reads for samples of *T. nylanderi* before and after filtering and trimming.

Sample Name	Raw	After Filtering	After Trimming
Infected 1	20137031	16292700	16128333
Nestmate 1	22373456	22246217	22047691
Infected 2	22123141	17241197	17075944
Nestmate 2	21099707	20980825	20787776
Infected 3	20648553	16792723	16620736
Nestmate 3	22546820	22402449	22190647
Infected 4	20619875	17573458	17396553
Nestmate 4	21281019	21157983	20948582
Nurse 1	15802933	15688999	15567444
Nurse 2	17235490	17105318	16967795
Nurse 3	15415218	15305925	15189042
Nurse 4	16176912	16082921	15977899
Queen 1	17068572	16977950	16844865
Queen 2	15456407	15333054	15216143
Queen 3	15730397	15648546	15528482
Queen 4	14401901	14307941	14193164

Table S9 Assembly statistics for the transcriptome of *T. nylanderi.*

Parameter	Value
Number of transcripts	38343
Mean length	3178.47
GC%	43
N50	4728

Table S10 Proteins involved in the melanisation pathway in *Drosophila melanogaster* (De Gregorio et al., 2001).

**Protein**	**Function**
serine protease	Activates prophenoloxidase (An et al., 2013)
serpin	Regulation of prophenoloxidase (De Gregorio et al., 2002)
(pro)phenoloxidase	Catalyze production of melanin (Dudzic et al., 2015)
Dopa decarboxylase (Ddc)	Converts dopa to dopamine (Nappi et al., 1992)
Tyrosinehydroxylase	Catalyzes tyrosine to dopa (Hultmark, 1993)
GTP cyclohydrolase (Punch)	Hydrolyses GTP (De Gregorio et al., 2001)
Dihydropteridine reductase (Dhpr)	Reduction of quinonoid dihydropteridine (Park et al., 2000)

Table S11 Search terms used for word search analysis based on the functional annotation using the UniProt database.

**Longevity**	**Fecundity**	**Immunity**	**Stress**
Toll	fecund	inflamm	stress
toll	fertil	immun	stressor
oxidative stress	meiosis	Toll	
TOR	meiotic	toll	
mTOR	zygot	defense	
tumor repressor	reproduct	bacteri	
UV damage	embryo	virus	
DNA repair	pregnancy	viral	
aging	mating	pathogen	
autophagy	fetal	infect	
senescence	parturition	Imd	
apopto	sexual		
lifespan	brood		
age-	egg		
homeosta	ovul		
transposable	ovary		
transposon	ovarian		

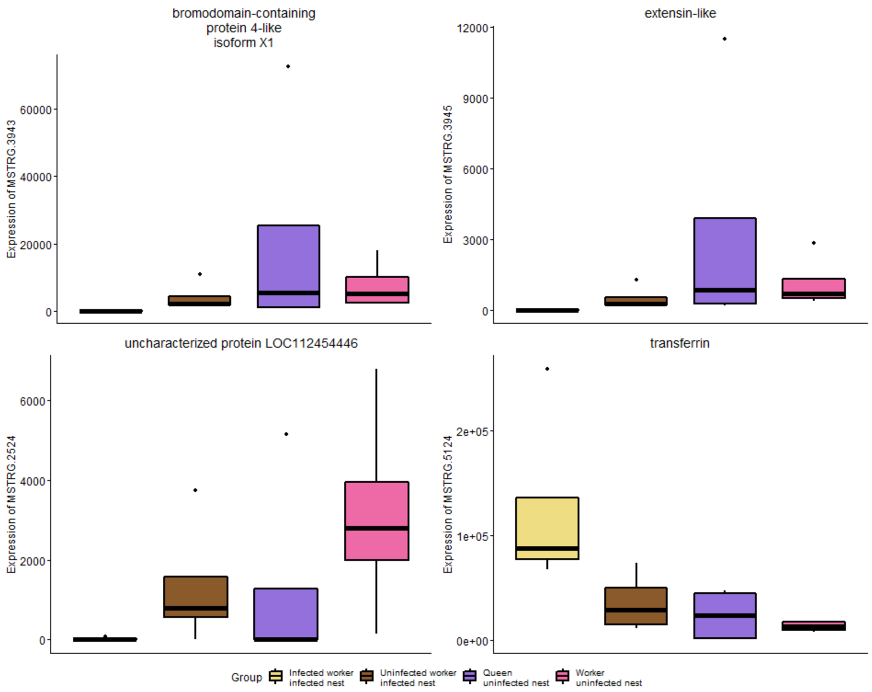

Figure S1 Expression of top four genes contributing to PC2 in the Principle Component Analysis.

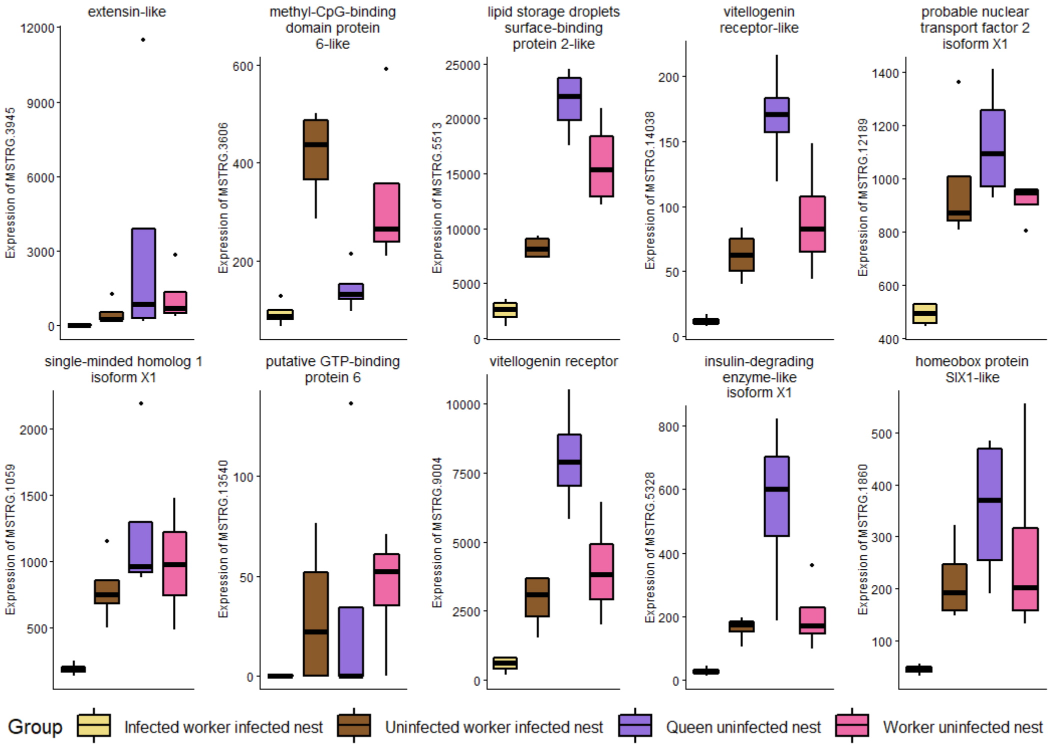

Figure S2 Top ten genes according to adjusted p-value that had a functional BLAST annotation upregulated in uninfected nestmates compared to infected workers of the same nest.

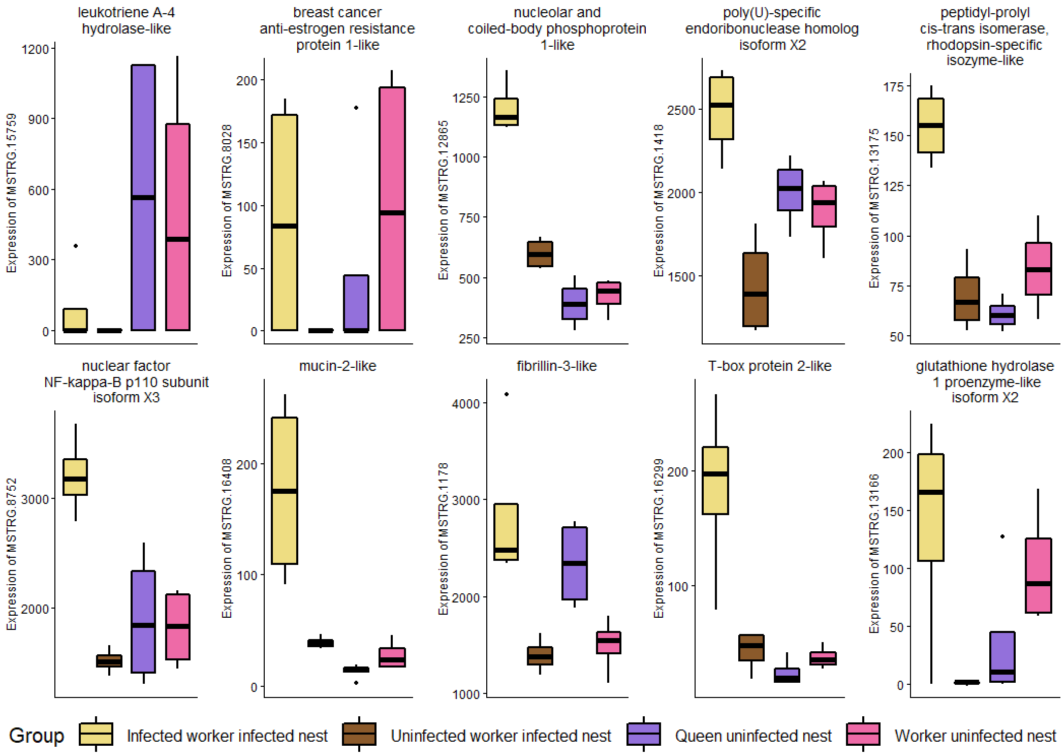

Figure S3 Top ten genes according to adjusted p-value that had a functional BLAST annotation upregulated in infected workers compared to their uninfected nestmates.

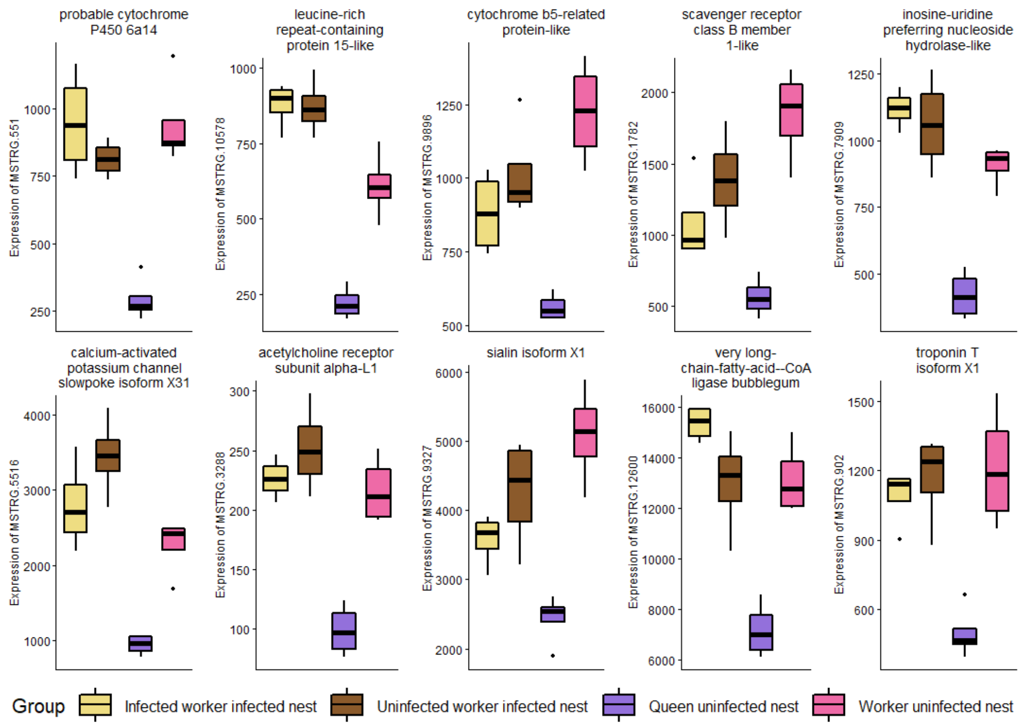

Figure S4 Top ten genes according to adjusted p-value that had a functional BLAST annotation upregulated in nurse compared to queens.

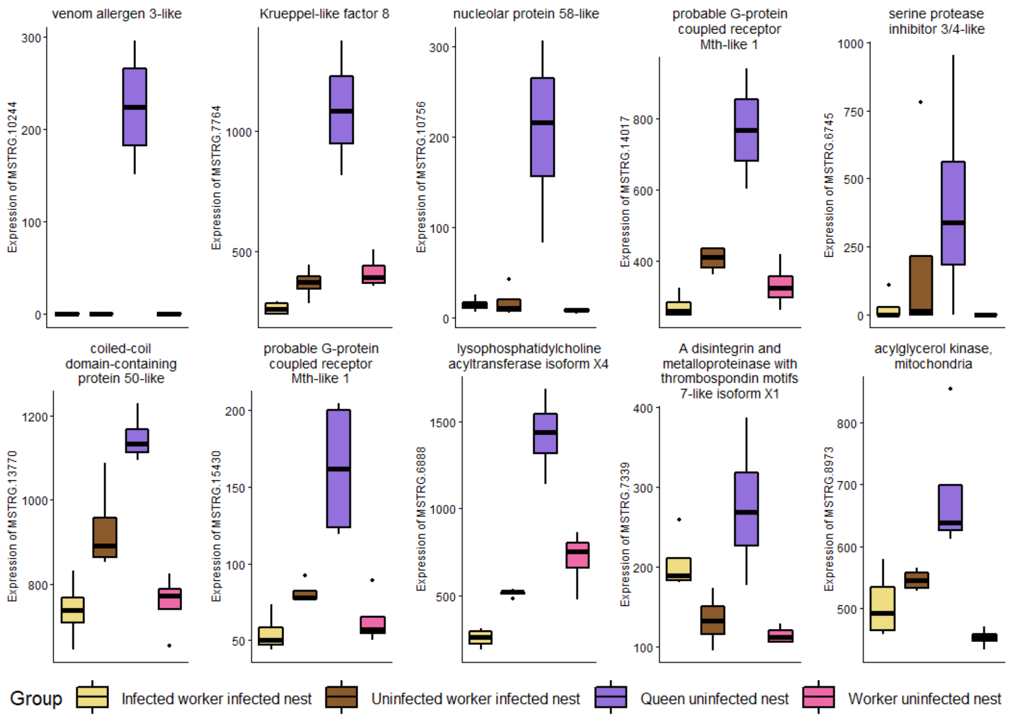

Figure S5 Top ten genes according to adjusted p-value that had a functional BLAST annotation upregulated in queens compared to nurses.

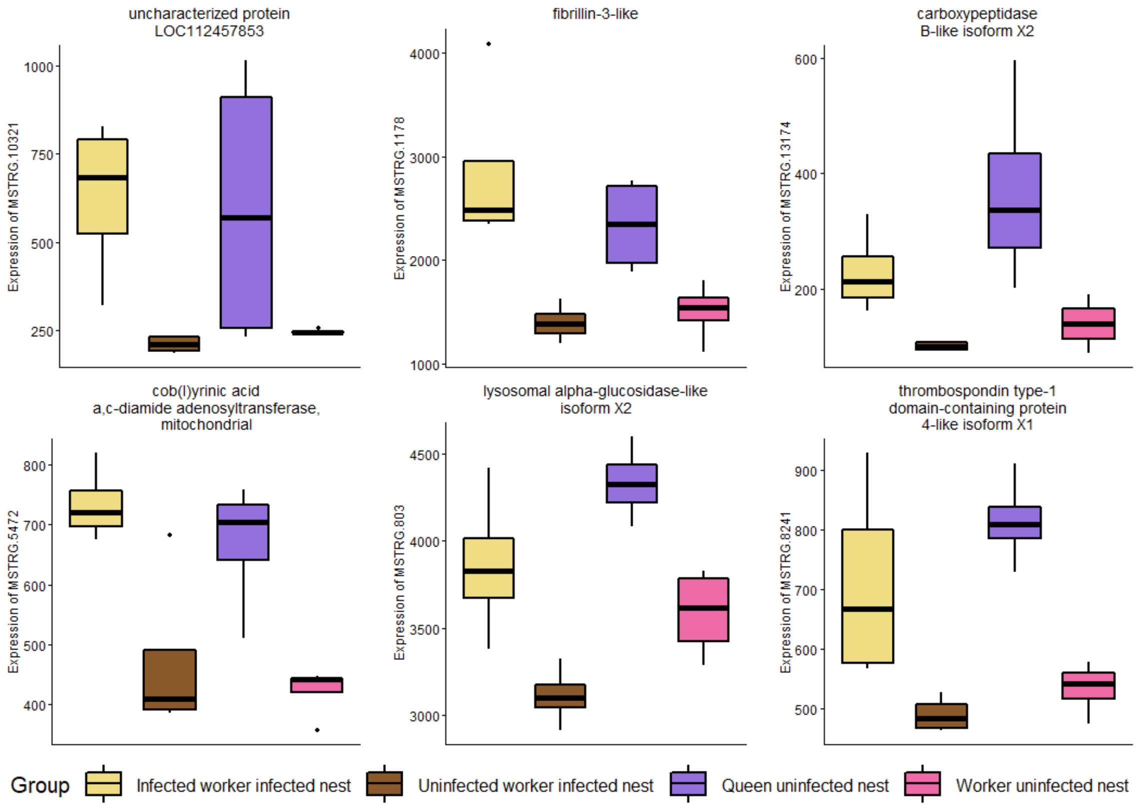

Figure S6 Genes overlapping between DEGs upregulated in queens and infected workers.

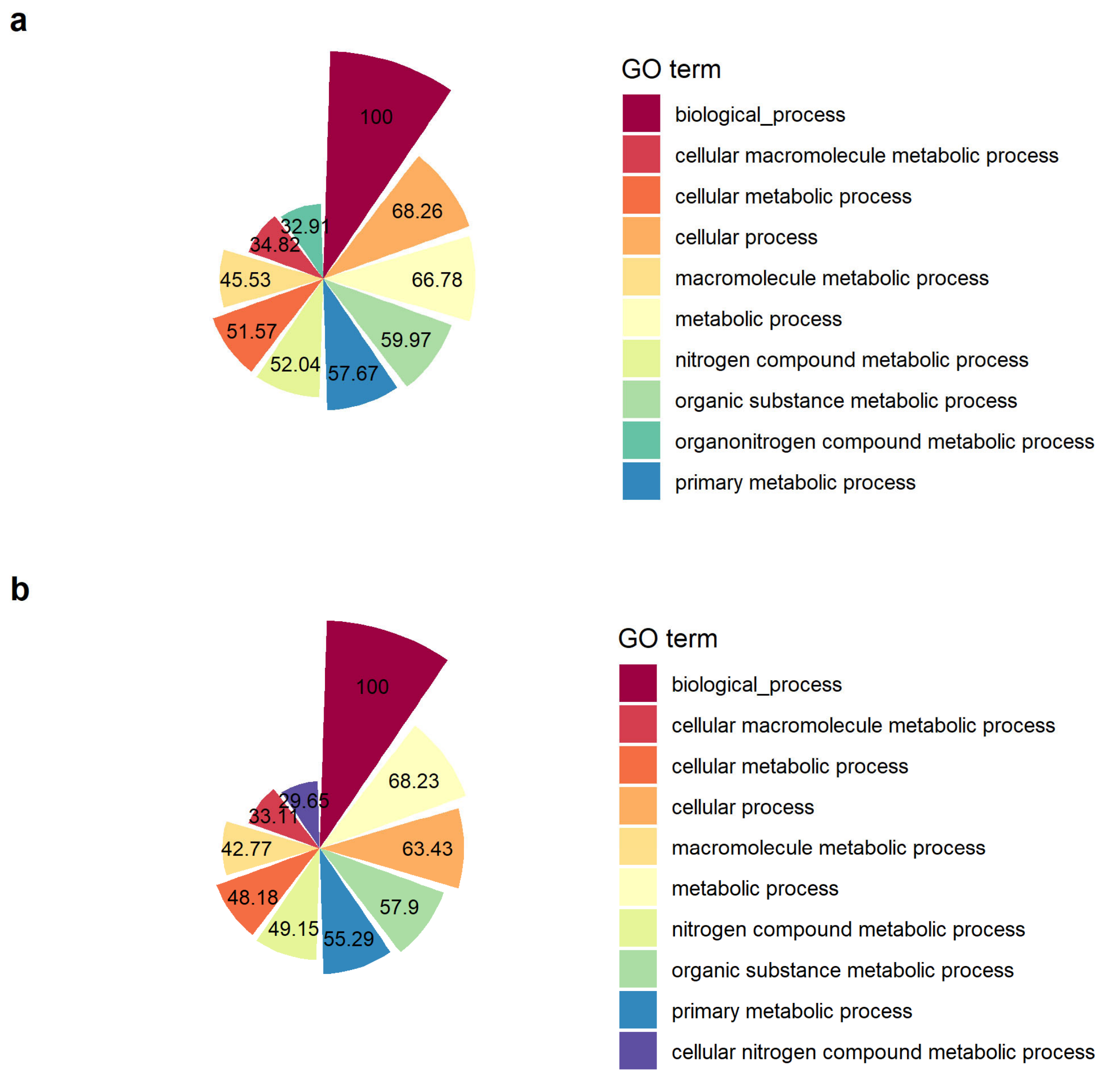

Figure S7 Ten most prevalent GO terms found in the cestode transcriptome (a) and the ant transcriptome (b). Percentage of GO annotations in respect to the total number of GO terms of "Biological Process" given in percent.

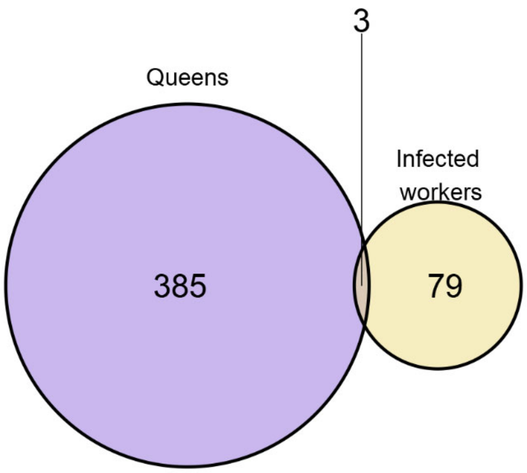

Figure S8 Overlap between differentially expressed genes upregulated in queens after controlling for colony size as batch effect (purple) and the genes upregulated in infected workers (yellow).

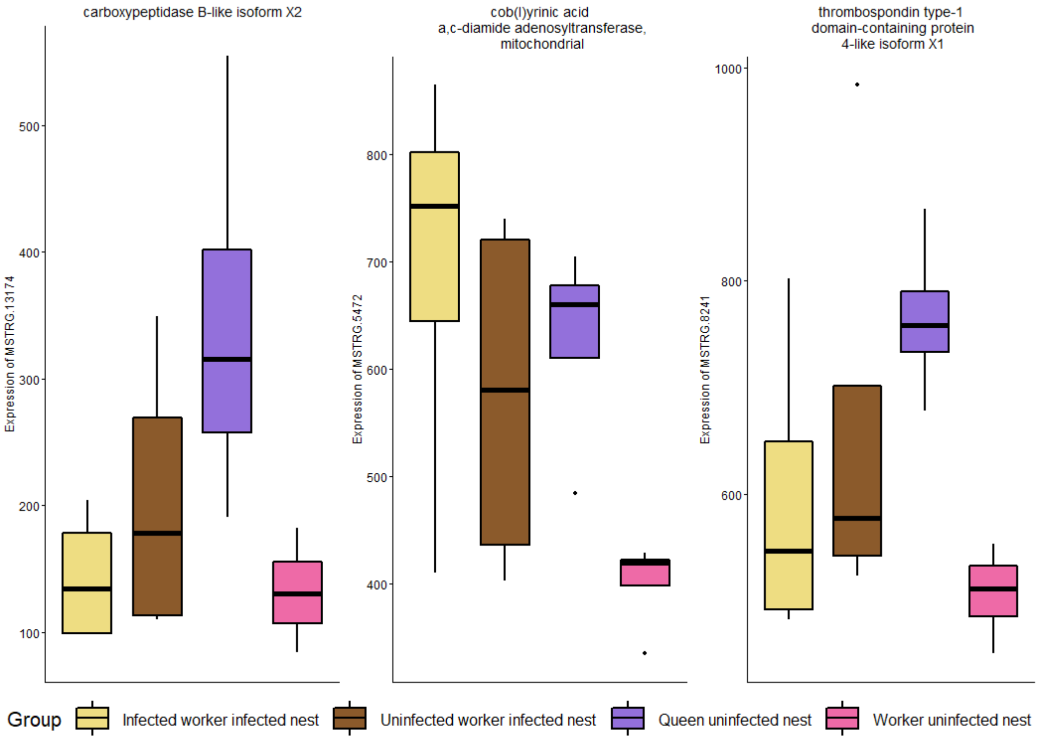

Figure S9 Genes and their expression in overlap between differentially expressed genes upregulated in queens after controlling for colony size as batch effect and the genes upregulated in infected workers (see Figure S8).

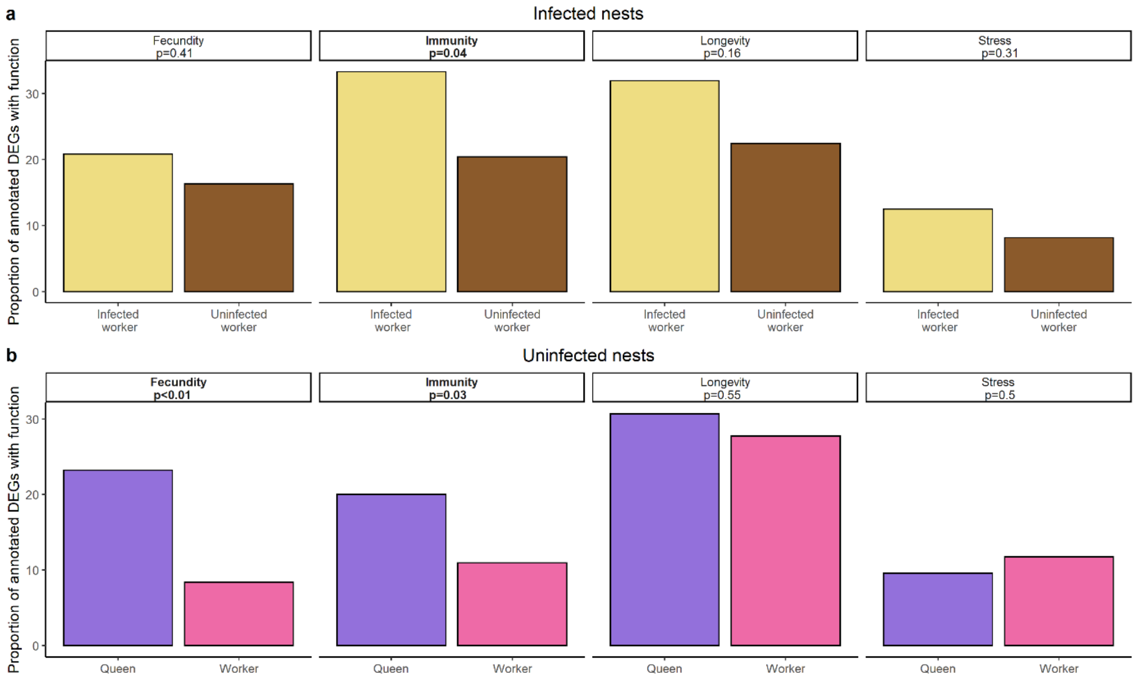

Figure S10 Results of text mining approach of terms related to fecundity, immunity, longevity and stress based on a UniProt search of upregulated genes in a: infected workers (yellow) and their uninfected nestmates (brown) of infected nests. b: queens (pink) and workers (purple) of uninfected nests when controlling for colony size as batch effect. P-value of χ2-test and the according function are given in boxes.

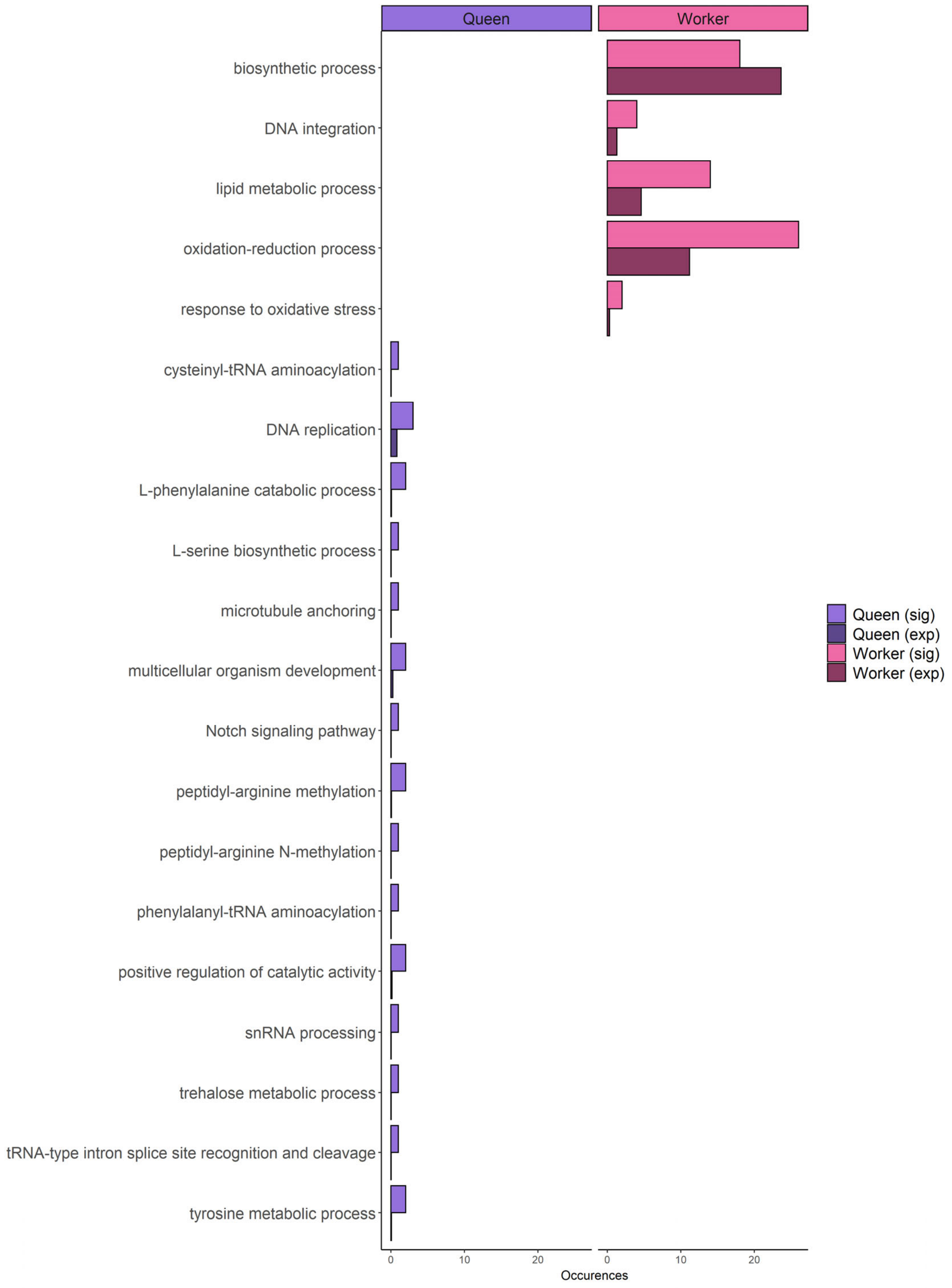

Figure S11 Bar plots depicting the significantly enriched Gene Ontology terms upregulated in ants from unparasitized nests: queens (left panel) and workers (right panel) after contolling for colony size as batch effect. Number of genes annotated with the specific term found in the candidate gene list are depicted (sig) as well as the number expected in a list of this size based on the annotation of the whole transcriptome (exp).

References

Afgan, E., Baker, D., Batut, B., Van Den Beek, M., Bouvier, D., Ech, M., Chilton, J., Clements, D., Coraor, N., Grüning, B.A., Guerler, A., Hillman-Jackson, J., Hiltemann, S., Jalili, V., Rasche, H., Soranzo, N., Goecks, J., Taylor, J., Nekrutenko, A., Blankenberg, D., 2018. The Galaxy platform for accessible, reproducible and collaborative biomedical analyses: 2018 update. Nucleic Acids Res. 46, W537–W544.

Altschul, S.F., Gish, W., Miller, W., Myers, E.W., Lipman, D.J., 1990. Basic local alignment search tool. J. Mol. Biol. 215, 403–410.

An, C., Zhang, M., Chu, Y., Zhao, Z., 2013. Serine protease MP2 activates prophenoloxidase in the melanization immune response of Drosophila melanogaster. PLoS One 8.

Bolger, A., Lohse, M., Usadel, B., 2014. Trimmomatic: a flexible trimmer for Illumina sequence data. Bioinformatics 30, 2114–2120.

De Gregorio, E., Han, S.J., Lee, W.J., Baek, M.J., Osaki, T., Kawabata, S.I., Lee, B.L., Iwanaga, S., Lemaitre, B., Brey, P.T., 2002. An immune-responsive Serpin regulates the melanization cascade in Drosophila. Dev. Cell 3, 581–592.

De Gregorio, E., Spellman, P.T., Rubin, G.M., Lemaitre, B., 2001. Genome-wide analysis of the Drosophila immune response by using oligonucleotide microarrays. Proc. Natl. Acad. Sci. U. S. A. 98, 12590–12595.

Dudzic, J.P., Kondo, S., Ueda, R., Bergman, C.M., Lemaitre, B., 2015. Drosophila innate immunity: Regional and functional specialization of prophenoloxidases. BMC Biol. 13.

Haas, B.J., Papanicolaou, A., Yassour, M., Grabherr, M., Blood, P.D., Bowden, J., Couger, M.B., Eccles, D., Li, B., Lieber, M., Macmanes, M.D., Ott, M., Orvis, J., Pochet, N., Strozzi, F., Weeks, N., Westerman, R., William, T., Dewey, C.N., Henschel, R., Leduc, R.D., Friedman, N., Regev, A., 2013. De novo transcript sequence reconstruction from RNA-seq using the Trinity platform for reference generation and analysis. Nat. Protoc. 8, 1494–1512.

Hultmark, D., 1993. Immune reactions in Drosophila and other insects: a model for innate immunity. Trends Genet.

Jones, P., Binns, D., Chang, H.Y., Fraser, M., Li, W., McAnulla, C., McWilliam, H., Maslen, J., Mitchell, A., Nuka, G., Pesseat, S., Quinn, A.F., Sangrador-Vegas, A., Scheremetjew, M., Yong, S.Y., Lopez, R., Hunter, S., 2014. InterProScan 5: Genome-scale protein function classification. Bioinformatics 30, 1236–1240.

Langmead, B., Salzberg, S.L., 2012. Fast gapped-read alignment with Bowtie 2. Nat. Methods 9, 357–359.

Li, B., Dewey, C.N., 2011. RSEM: Accurate transcript quantification from RNA-Seq data with or without a reference genome. BMC Bioinformatics 12, 323.

Nappi, A.J., Carton, Y., Li, J., Vass, E., 1992. Reduced cellular immune competence of a temperature-sensitive dopa decarboxylase mutant strain of *Drosophila* melanogaster against the parasite Leptopilina boulardi. Comp. Biochem. Physiol. -- Part B Biochem. 101, 453–460.

Park, D., Park, S., Yim, J., 2000. Molecular characterization of Drosophila melanogaster dihydropteridine reductase. Biochim. Biophys. Acta - Gene Struct. Expr. 1492, 247–251.

Simão, F.A., Waterhouse, R.M., Ioannidis, P., Kriventseva, E. V., Zdobnov, E.M., 2015. BUSCO: Assessing genome assembly and annotation completeness with single-copy orthologs. Bioinformatics 31, 3210–3212.

Smith-Unna, R., Boursnell, C., Patro, R., Hibberd, J.M., Kelly, S., 2016. TransRate: Reference-free quality assessment of de novo transcriptome assemblies. Genome Res. 26, 1134–1144.

Wingett, S.W., Andrews, S., 2018. FastQ Screen: A tool for multi-genome mapping and quality control. F1000Research 7, 1338.
